# The complete chloroplast genome of *Firmiana danxiaensis*, an endangered species endemic to Danxia landform in Southern China

**DOI:** 10.1080/23802359.2019.1689861

**Published:** 2019-11-18

**Authors:** Qifeng Lu, Wenhua Luo, Zhihuan Huang

**Affiliations:** a Guangxi Institute of Botany, Guangxi Zhuang Autonomous Region and Chinese Academy of Sciences, Guilin, PR China;; b School of Architecture, University of South China, Hengyang, PR China

**Keywords:** *Firmiana danxiaensis*, chloroplast genome, phylogenetic analysis

## Abstract

We sequenced the complete chloroplast of *Firmiana danxiaensis* (Malvaceae), which is 161,253 bp in size and consists of a large single-copy region (LSC) of 90,142 bp and a short single copy region (SSC) of 20,067 bp. It was separated by two inverted repeats (IRs) regions of 25,522 bp for each. The GC content of the whole genome, LSC, SSC, and IRs region was 36.87%, 34.68%, 31.24%, and 42.97%, respectively. The overall base content was A (31.07%), T (32.05%), C (18.80%), and G (18.08%). The genome contained 127 genes, including 82 protein-coding sequences, 37 tRNA genes, and 8 rRNA genes. Phylogenetic analysis showed that *F. danxiaensis* is sister to *F. simplex*, and supported there was a close relationship among *F. danxiaensis*, *F. simplex,* and *F. pulcherrima*.

*Firmiana danxiaensis* (Malvaceae) is a threatened tree species that was included in a Second Class Protected Key Wild Plant of China and the China Species Red List. It is considered as a dominant species and distributed only in Danxia Mountain, Guangdong Province, China. This tree species has been the focus of field investigation, community characters, and population genetic studies over the last 30 years by researchers (Fan et al. 2013; Chen et al. 2014; Chen et al. 2015). However, the chloroplast (cp) genome information and systematics of *Firmiana* genus remains unclear. Therefore, in this study, we reported the whole cp genome of *F. danxiaensis* based on high-throughput sequencing, and reconstructed the phylogeny to other related species of *Malvaceae* species with the whole cp genome sequences.

One seedling of *F. danxiaensis* was collected from Danxia Mountain, Guangdong Province, China, and was planted in Guilin botanic garden (Guilin, China; 25°04′26″N, 110°17′46″E, 165 m a.s.l.). Two years later, fresh leaves of the seeding were collected to extract the cp DNA by modified CTAB method (Doyle 1987). The extracted DNA was sequenced using Illumina NovaSeq platform at Genepioneer Biotechnologies Inc. (Nanjing, China). Approximately 19.73 million pair-end reads (2 × 150 bp) were generated. The cp genome reads of the *F. danxiaensis* were assembled by using SPAdes version 3.10.1 (Anton et al. 2012), and gaps were filled by Gapfiller version 2.1.1 and polymerase chain reaction (PCR)-base methods. The assembled cp genome was compared with the reference cp genome of *Gossypium thurberi* (GU907100.1). The annotation of cp genome sequence was performed by using BLAST version 2.2.25 (https://blast.ncbi.nlm.nih.gov/Blast.cgi), rRNAs were predicted through HMMER version 3.1b2 (http://www.hmmer.org/), and tRNA was identified through aragorn version 1.2.38 (http://130.235.244.92/ARAGORN/). The cp genome map was generated by using OGDRAW (https://chlorobox.mpimp-golm.mpg.de/OGDraw.html).

The cp genome of *F. danxiaensis* is 161,253 bp in length and has a typical quadripartite circular structure, consisting a large single-copy (LSC) region of 90,142 bp, a short single-copy (SSC) region of 20,067 bp, and a pair of inverted repeats (IRs) (IRa and IRb, 25,522 bp each). The cp genome of *F. danxiaensis* base content of A, T, C, and G was 31.07%, 32.05%, 18.80%, and 18.08%, respectively. The GC content of the cp genome was 36.87%, and SSC (31.24%) had a lower GC content than LSC (34.68%), and IRs (42.97%) region, which is similar to *F. major* (Ya et al. 2018). The cp genome including 56.4% coding regions, consists of 127 genes (including 82 protein-coding sequences, 37 tRNA genes, and 8 rRNA genes). The non-coding region includes 10.2% introns and 33.4% intergenic spacers. Fifteen genes (*tRNA-UUU*, *tRNA-CGA*, *atpF*, *rpoC1*, *tRNA-UAA*, *tRNA-ACA*, *rpl2*, *ndhB*, *tRNA-UUC*, *tRNA-UGC*, *ndhA*, *tRNA-UGC*, *tRNA-UUC*, *ndhB*, and *rpl2*) had one intron and two genes (*ycf3* and *clpP*) had two introns. Seventeen genes (including six protein-coding genes, seven tRNAs, and four rRNAs) were duplicated in IRs region.

To explore the phylogenic relationships of *F. danxiaensis* among Malvaceae family, we selected 13 published cp genome of Malvaceae species and *Lupinus albus* as an outgroup. The Maximum Likelihood (ML) tree was reconstructed by RAxML version 8.2.10 (Stamatakis et al. 2008). The ML tree showed that *F. danxiaensis* is sister to *F. simplex* (Figure 1), and supported there was a close relationship among *F. danxiaensis*, *F. simplex,* and *F. pulcherrima*, but a relatively distant relationship to *F. major*.

**Figure 1.**
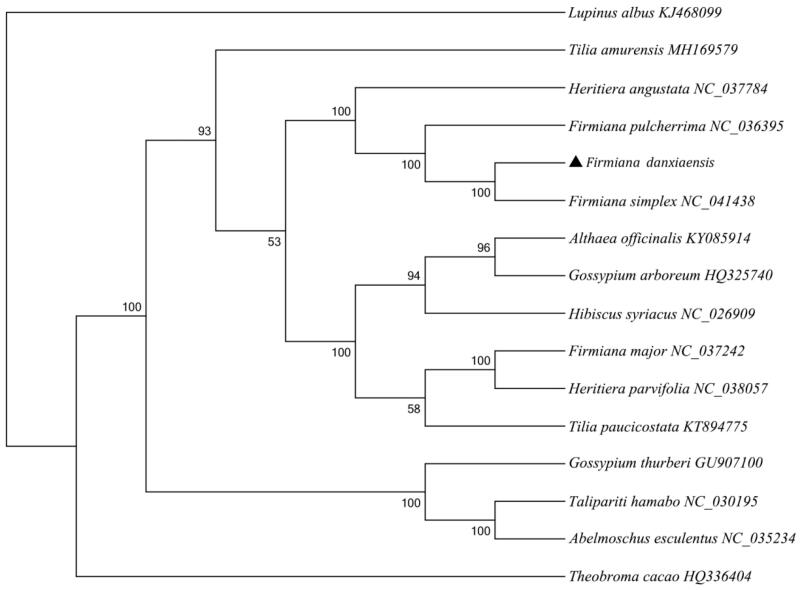
Phylogenetic tree was constructed by using Maximum Likelihood (ML), based on the whole chloroplast genome of *F. danxiaensis* and its related species. Numbers indicate bootstrap values from maximum likelihood analyses.
